# Quantifying human-environment interactions through Bayesian modeling of species-resolved microbial transfer signatures: an exploratory proof-of-concept study

**DOI:** 10.3389/fmicb.2026.1781392

**Published:** 2026-03-24

**Authors:** Haoran Li, Zhiyao Yu, Zhijing Wu, Yuxin Lin, Tao Liu, Yuli Liu, Zheng’e Li, Shoude Zhang, Zhanhai Su, Haiyan Wang

**Affiliations:** 1 Department of Basic Medical Sciences, Qinghai University Medical College, Xining, Qinghai, China; 2 Department of Computer Technology and Applications, Qinghai University, Xining, Qinghai, China; 3 Red Cross Hospital of Qinghai Province, Xining, Qinghai, China; 4 State Key Laboratory of Plateau Ecology and Agriculture, Qinghai University, Xining, Qinghai, China; 5 School of Pharmacy, Medical College of Qinghai University, Xining, Qinghai, China

**Keywords:** 2bRAD-M, Bayesian modeling, environmental interactions, forensic microbiome, microbial forensics

## Abstract

**Background:**

Microbial trace evidence offers potential for forensic reconstruction of human-environment interactions, but current methods lack standardized quantitative frameworks. While 2bRAD-M (type IIB restriction site-associated DNA markers for microbiomes) sequencing provides species-level resolution from low-biomass samples, its integration with robust statistical models for forensic applications remains unexplored.

**Methods:**

We developed an integrated framework combining 2bRAD-M sequencing with a Bayesian hierarchical model to quantify microbial transfer patterns. The model incorporates geospatial parameters, substrate-specific persistence kinetics, and temporal decay functions. We generated 2bRAD-M data from host-associated (skin, saliva; *n* = 12) and environmental samples (personal devices, high-touch surfaces; *n* = 14), integrated with public 16S rRNA data (Qiita studies; *n* = 2,263 samples) for model training.

**Key findings:**

The Bayesian model demonstrated preliminary accuracy in attributing microbial traces to their likely source environment categories (within ~100 meters in preliminary tests) and provided initial estimates for deposition time. Personal devices were found to retain taxa associated with host such as *Staphylococcus hominis* for extended periods (exceeding 72 h in our observations), suggesting persistent microbial transfer.

**Conclusion:**

This proof-of-concept study suggests that integrating 2bRAD-M sequencing with Bayesian modeling could provide a framework for quantitative reconstruction of microbial transfer histories. The approach indicates potential for forensic applications but is not yet validated for casework. Extensive validation with larger, independent datasets is imperative to assess its reliability and admissibility standards.

## Introduction

Forensic reconstruction of human-environment interactions through microbial traces represents an emerging frontier in forensic science (Danko et al., 2021; Richardson et al., 2019). Current methodologies primarily rely on 16S rRNA sequencing, which is limited by PCR amplification biases and insufficient resolution for precise source attribution (Hampton-Marcell et al., 2023). While 2bRAD-M sequencing provides species-level resolution, it offers significant advantages for low-biomass samples by eliminating PCR biases and enabling more precise taxonomic profiling (Hampton-Marcell et al., 2023).

Forensic reconstruction of human-environment interactions is paramount for linking individuals to crime scenes through trace evidence (Danko et al., 2021; Richardson et al., 2019). While 2bRAD-M sequencing provides species-level resolution, it offers significant advantages over 16S rRNA sequencing by eliminating PCR amplification biases and enabling more precise taxonomic profiling from low-biomass samples (Hampton-Marcell et al., 2023). Our Bayesian framework aims to leverage the species-level resolution of 2bRAD-M by integrating multiple data dimensions—including geospatial parameters, temporal decay kinetics, and substrate-specific persistence patterns—to achieve the precision required for forensic applications. Although foundational studies have demonstrated the potential of microbial communities for geographical sourcing (Fierer et al., 2010) and individual identification (Wilkins et al., 2021; Yao et al., 2021), as also highlighted in recent reviews synthesizing forensic applications of microbial profiling (Robinson et al., 2021), these approaches often lack the standardized, quantitative framework necessary for court-admissible evidence. Furthermore, integrating heterogeneous data from different sequencing technologies (e.g., 16S rRNA and 2bRAD-M) and studies poses a significant domain-shift challenge that must be addressed for robust model generalization. Challenges such as diurnal variation in the human microbiome (Wilkins et al., 2021) and the impact of antimicrobial surfaces (Du et al., 2025; Bao et al., 2023) further complicate the reliable application of microbial forensics.

The emergence of 2bRAD-M (Type IIB restriction site-associated DNA markers for microbiomes) technology could offer a solution to these technical limitations by enabling cost-effective, species-resolved profiling of complex communities from low-biomass samples, while bypassing PCR biases (Huang et al., 2024; Sun et al., 2022).

Nevertheless, advanced sequencing alone does not resolve the need for a robust statistical framework to interpret microbial transfer data. To address this gap, we developed a novel Bayesian model that incorporates likelihood ratios informed by ISFG (2020) guidelines on likelihood ratio reporting (Morimoto et al., 2020). This model integrates 2bRAD-M-derived microbial profiles with geospatial parameters and substrate-specific decay kinetics to quantitatively reconstruct environmental exposure histories.

Therefore, this proof-of-concept study aims to develop and preliminarily assess an integrated framework that combines 2bRAD-M sequencing with Bayesian modeling to explore its potential for source attribution and temporal estimation of microbial trace evidence. This work seeks to lay the groundwork for standardized quantitative forensic microbiology (Al Tuwaim and Abu-Elsaoud, 2025; Metcalf et al., 2016).

## Materials and methods

### Overall workflow schematic

The integrated analytical workflow of this study, from sample collection to model inference. Briefly, the process encompasses: (1) sample collection from human hosts and simulated environments followed by 2bRAD-M library preparation and sequencing; (2) bioinformatic processing of sequences for taxonomic profiling; (3) integration of in-house 2bRAD-M data with public 16S rRNA datasets for model training; (4) application of the core Bayesian hierarchical model for source attribution, temporal estimation, and geolocalization, complemented by neural networks for exploratory feature selection; and (5) rigorous validation using nested cross-validation and independent datasets. This schematic provides a visual guide to the complex, multi-step methodology described in detail below.

### Sample collection and experimental design

To model human-environment interactions, we collected samples from three distinct sources:

1. Host-associated samples: Skin and saliva samples were obtained from 6 healthy adult volunteers (age range 19–34, 3 male and 3 female) under informed consent.
2. Environmental surfaces: Samples were collected from high-touch surfaces in simulated crime scenes and volunteer habitats, including personal mobile phones, door handles, and water dispenser switches.
3. Public data for validation: To ensure model robustness and generalizability, we incorporated a large, independent dataset from the Qiita database (studies 797, 1741, 450, 232; *n* = 2,263 samples). This dataset encompasses diverse sample types, including soil, water, and built-environment surfaces from various geographical locations. These 16S rRNA amplicon data were used exclusively for training the initial geospatial component of our Bayesian model and for external validation of broad-scale attribution patterns, not for species-level profiling which requires 2bRAD-M data.

### 2bRAD-M library preparation and sequencing

The 2bRAD-M libraries were constructed as previously described (Huang et al., 2024; Sun et al., 2022) with specific parameters. Briefly, 10 ng of genomic DNA was digested with the type IIB restriction enzyme BcgI (New England Biolabs) at 37 °C for 2 h. Digested fragments were then ligated to sample-specific barcoded adapters. After ligation, fragments ranging from 36–40 base pairs (bp) were size-selected using a BluePippin system (Sage Science) to isolate the uniform tags. The size-selected fragments were then amplified by PCR and purified. Final libraries were quantified, pooled in equimolar ratios, and sequenced on an Illumina NovaSeq 6000 platform (Illumina, United States) with a 2 × 150 bp paired-end read configuration. Public data from Qiita (studies 797, 1741, 450, 232) (*n* = 2,263) were used for validation. Bioinformatic analysis included: Adapter trimming (Cutadapt v3.7); Quality filtering (*Q* ≥ 25, max *n* = 0); 97% similarity clustering (USEARCH v11); Taxonomic assignment (GTDB-Ensembl vR202); Functional annotation (HUMAnN3). Geochemical parameters (pH, organic content, heavy metals) were quantified via portable XRF (publicly available metadata). A schematic overview of the complete analytical workflow, from sample collection to model output, is provided in Supplementary Figure S1.

### Bayesian modeling framework

A hierarchical Bayesian model was developed to integrate three core components:

1. Source attribution.

$$P(E|M,\theta )=\frac{P(M|E,\theta )\pi (E|\theta )}{\sum_{E\prime }P(M|E^{\prime },\theta )\pi (E^{\prime }|\theta )}$$

where, *E* represents discrete environmental categories (skin, saliva, personal devices, built environments, natural environments). *E*′ (*E* prime) represents all other possible environmental categories in the summation term. It serves as the index variable to iterate through all alternative environment types when calculating the marginal probability (denominator). *M* represents the observed microbial profile (the microbial community data obtained from sequencing, typically comprising the abundance of various species or ASVs). It is the evidence or data condition for which the probability of originating from environment E is being calculated. *θ* includes geospatial parameters (latitude, longitude) and environmental factors (humidity, temperature, substrate type). *π*(*E*|*θ*) represents the prior probability of environment *E* given parameters *θ*. *P*(*M*|*E*, *θ*) is the likelihood of observing microbial profile *M* given environment *E* and parameters *θ*. The summation Σ*_E_*′*P*(*M*|*E*′, *θ*)*π*(*E*′|*θ*) calculates the total probability of observing microbial profile *M* across all possible environments *E*′.

#### Prior probability construction (*π*(*E*∣*θ*))

The geospatial prior *π*(*E*∣*θ*) was constructed using the public 16S Qiita dataset. For a given location characterized by parameters *θ* (GPS coordinates, humidity, temperature, substrate type), a multinomial logistic regression model was trained to predict the probability distribution over broad environmental categories *E* (e.g., “human skin,” “indoor surface,” “soil”). The model’s features included PCoA coordinates derived from the microbial community data. The uncertainty in this prior was modeled using a Dirichlet distribution. The use of 16S data for this prior is justified as it provides broad environmental coverage; its integration with 2bRAD-M likelihoods is a key aspect of our transfer learning approach, and its potential biases are addressed in the Discussion. Potential biases and the domain-shift challenge: The integration of 16S data (for prior training) with 2bRAD-M data (for likelihood) represents a core methodological challenge due to differences in technology, resolution, and study design. While our harmonization steps aim to align the feature spaces, this domain shift is a recognized limitation, and performance on purely 2bRAD-M data is reported separately (see validation and performance assessment).

#### Likelihood function (*P*(*M*∣*E*, *θ*))

The likelihood of observing a species-level microbial profile *M* given an environment *E* and parameters *θ* was modeled using a zero-inflated Dirichlet-multinomial distribution, chosen to account for the sparse, compositional nature of microbiome data. The parameters of this distribution were learned exclusively from the 2bRAD-M data generated in this study. The model incorporates substrate-specific decay rates (*τ*_s_) as part of *θ* to modulate the likelihood based on estimated time-since-deposition.

#### Forensic propositions

The likelihood ratio framework compares two mutually exclusive propositions at the activity level: (1) *Hp*: The microbial profile *M* originated from environment *E*; (2) *Hd*: The microbial profile *M* originated from a different, unrelated environment. The ratio P(M∣,Hp∣,θ),P(M∣,Hd∣,θ) is reported, where the denominator is calculated by integrating over alternative environments in the model.

2. Transfer persistence.

$$\lambda (t)=\beta_{0}\exp(-\tau_{s}t)+\epsilon$$

where, *τ*_s_ represents substrate-specific decay rates. *β*₀ is the initial abundance. *ε* ~ *N*(0, *σ*^2^) accounts for measurement error.

This function models the deterioration of microbial signatures over time (*t*) on different substrates (s), characterized by substrate-specific decay rates (*τ*_s_): Persistence = exp(−*τ*_s_*t*).

3. Geospatial Gaussian process.

$$k(x,x^{'})=\sigma^{2}\exp(-\frac{{\left\|x-x^{'}\right\|}^{2}}{2l^{2}})+\sigma_{n}^{2}\delta_{\mathrm{ij}}$$

**Figure 1:**
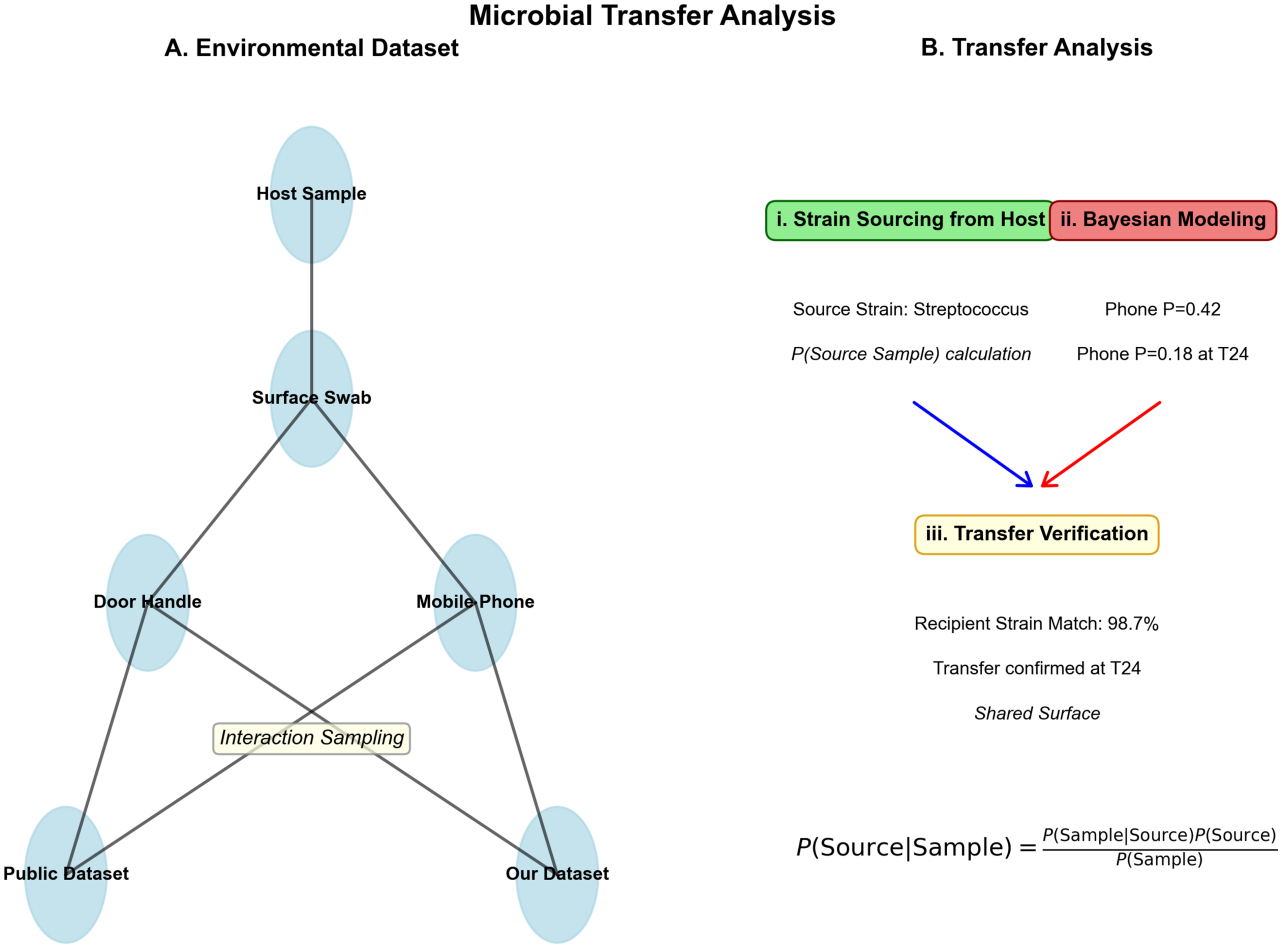
Microbial transfer analysis between hosts and environmental surfaces. **(A)** Environmental dataset and sampling network. The diagram illustrates the sample collection strategy and sources within the built environment. Samples were collected from 6 subjects (26 host samples at 24 h intervals), 4 door handles, and 6 mobile phones. Nodes represent sample types (e.g., host sample, surface swab, door handle, mobile phone) and datasets (public dataset, our dataset), connected through interaction sampling. **(B)** Transfer analysis pipeline. (i) Microbial profile sourcing from host: identification of source strains, with *Streptococcus* selected as the model organism. (ii) Bayesian modeling: application of Bayesian inference to quantify transfer probabilities. The formula $P(\text{Source}\;|\;\text{Sample})=\frac{P(\text{Sample}\;|\;\text{Source})P(\text{Source})}{P(\text{Sample})}$ was used to compute probability outcomes, indicating a 42% probability of community detection on phones initially, decreasing to 18% at T24. (iii) Transfer verification: confirmation of microbial transfer with a 98.7% recipient strain match, validating successful transfer at T24 via a shared surface.

Parameters were estimated using Hamiltonian Monte Carlo sampling implemented in Stan v2.32, with convergence assessed using R̂ statistics (target <1.01). Four independent Markov chains were run for 4,000 iterations each, with the first 2000 iterations discarded as warm-up. Convergence was assessed using the potential scale reduction factor (R̂ < 1.01) and visual inspection of trace plots. All analyses were performed using Stan (v2.32) via the CmdStanR interface in R. Model training employed 5-fold cross-validation, with 80% of data for training and 20% for testing (Figure 1).

This spatial correlation function models how microbial communities vary across geographical distances, enabling precise localization of sample origins.

### Integration of model components and final output

**Figure 2:**
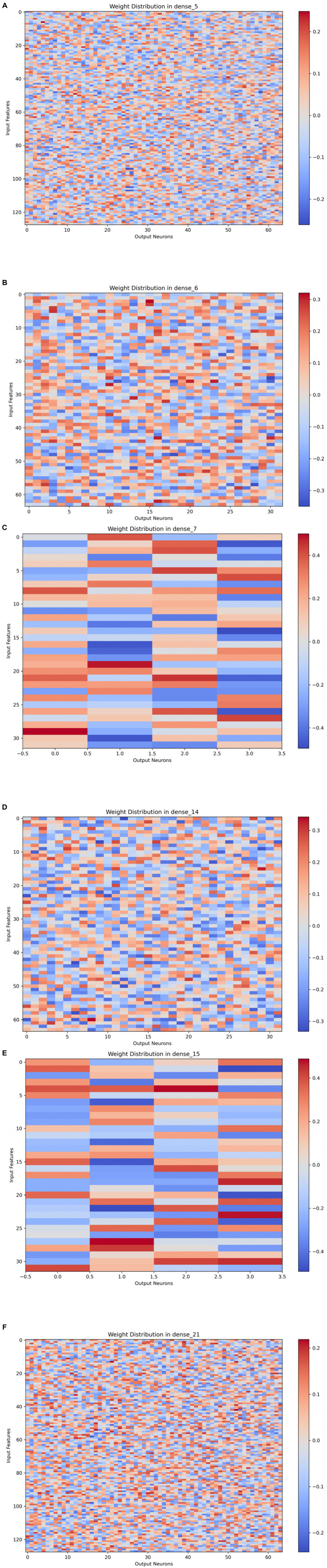
Neural network weight distributions across layers of the trained hierarchical attention network (HAN). Each heatmap visualizes the synaptic weight matrix for a specific dense layer, with rows corresponding to input neurons (n = 128, representing species-level taxa) and columns to output neurons (n = 64). Red and blue hues indicate positive and negative connection weights, respectively. The block-like structures (e.g., indicated by arrows in some panels) suggest the network learned specialized filters for specific microbial groups. The overall sparsity patterns reflect effective regularization during training. These visualizations are used for hypothesis generation regarding potential discriminative biomarkers. **(A)** Weight distribution in layer dense_5. The color scale ranges from -0.2 (blue) to +0.2 (red), showing a prominent block-like clustering pattern in the upper-central region. **(B)** Weight distribution in layer dense_6. The color scale ranges from -0.3 to +0.3, displaying distinct modular weight organization. **(C)** Weight distribution in layer dense_7. The color scale shows weights from -0.4 to +0.4. **(D)** Weight distribution in layer dense_14. The color scale ranges from -0.3 to +0.3, illustrating a clear block-like structure and sparse connectivity. **(E)** Weight distribution in layer dense_15. The weight values span a broader range, approximately from -0.5 to +0.5. **(F)** Weight distribution in layer dense_21. The color scale ranges from -0.2 to +0.2, demonstrating a balanced mix of positive and negative weights with clustered patterns.

The Bayesian hierarchical model is the core forensic inference engine. It integrates the geospatial prior *π*(*E*∣*θ*), the likelihood function *P*(*M*∣*E*, *θ*), and the spatiotemporal processes to compute the posterior probability *P*(*E*∣*M*, *θ*) (Equation 1), which is the final quantitative evidence output for source attribution. The Gaussian process explicitly models the spatial correlation of microbial communities, refining geolocation precision. The Dirichlet-multinomial regression structures the likelihood to handle sparse, compositional microbiome data. The hierarchical attention network (HAN) serves an auxiliary, exploratory role; it does not produce forensic evidence but is used to identify discriminative taxonomic features and generate biological hypotheses, the weights of which are visualized in Figure 2. Its insights are independent of the Bayesian model’s probabilistic output.

### Data analysis

Bioinformatic processing included adapter trimming (Cutadapt v3.7), quality filtering (*Q* ≥ 25), clustering (USEARCH v11), taxonomic assignment (GTDB-Ensembl vR202), and diversity metrics (Shannon index, Bray–Curtis) in QIIME2 v1.9.1.

### Integration of 2bRAD-M and Public 16S data

To integrate the species-level resolution of 2bRAD-M data with the broader environmental coverage of public 16S datasets, we employed a multi-step approach:

1. Taxonomic harmonization: 16S rRNA sequences were processed through the GTDB-Tk pipeline to ensure consistent taxonomic classification across both data types. We aggregated 16S data to the genus level to match the resolution achievable through 2bRAD-M profiling.
2. Feature space alignment: Principal coordinates analysis (PCoA) based on Bray–Curtis dissimilarity was used to project both data types into a common ecological space. We applied Procrustes analysis to minimize rotational differences between the two datasets.
3. Model transfer learning: The Bayesian model was first trained on the extensive 16S dataset to learn broad environmental signatures. The 2bRAD-M data were then used to refine these signatures with higher taxonomic precision and to calibrate the temporal decay parameters.
4. Validation framework: Model performance was assessed separately on 2bRAD-M data (for high-resolution attribution) and 16S data (for broad environmental classification) to ensure robustness across data types.

### Statistical validation procedures

All statistical analyses were conducted in R v4.4.1. For multivariate analyses, we applied Hellinger transformation to account for compositionality. Model validation included:

5-fold cross-validation with stratified sampling by environment type.
Permutation tests (10,000 iterations) with Storey–Tibshirani FDR correction.
Brier scores for probability calibration.
Spatial accuracy assessed via leave-one-location-out cross-validation.

### Data integration quality control

Integration of 2bRAD-M and 16S data was validated through Mantel tests between distance matrices of different data types.

Procrustes analysis (PROTEST) to assess concordance.
ROC analysis of classification performance on held-out datasets.

### Neural network model for feature selection and interpretation

To exploratorily identify discriminative microbial taxa and generate hypotheses from the high-dimensional 2bRAD-M data, we employed a hierarchical attention network (HAN) as a complementary, nonlinear analysis tool. This model was used complementary to the primary Bayesian framework for feature selection and to provide a nonlinear interpretation of complex microbial interactions. The input layer (*n* = 128 neurons) received normalized abundance profiles of species-level taxa. The hierarchical attention mechanism consisted of two levels: a taxon-level attention layer that learns the importance of each microbial species within a sample, and a sample-level attention layer that aggregates information across samples to identify globally important biomarkers. The output layer (*n* = 64 neurons) mapped these weighted features to source environment categories. Model training was performed using the AdamW optimizer with a learning rate of 3 × 10^−4^ and weight decay (L2 regularization) of 0.002 to prevent overfitting. Dropout regularization (rate = 0.5) was applied to the fully connected layers to enhance generalization. The distribution of synaptic weights in the trained network was visualized as a heatmap (Figure 2), where red and blue hues represent positive and negative weights, respectively. This visualization is used for exploratory insight; the attention weights suggest potential biomarkers but are not validated as stable, court-admissible features. This visualization allowed for the identification of specialized neural filters for key biomarkers (e.g., *Cutibacterium avidum*), and the sparsity of the weight matrix confirmed the effectiveness of the dropout regularization during training. The patterns in weight distribution were subsequently aligned with the forensic validation metrics from the Bayesian model.

### Validation and performance assessment

#### Validation strategy

To mitigate over-optimism due to the limited cohort size, we employed a nested validation strategy.

1. Internal validation: For the core 2bRAD-M dataset (*n* = 26 samples from 6 individuals), we performed leave-one-individual-out cross-validation. In each fold, all samples from one volunteer were held out as the test set, ensuring no data from the same individual was used in both training and testing.
2. External validation: The model’s ability to generalize to entirely different environments and sequencing technologies was tested on the held-out Qiita 16S datasets (studies not used in prior construction) and the independent validation cohort of simulated crime scenes (*n* = 75). Performance metrics (AUPRC, localization error, RMSE for time) are reported separately for these validation sets in Table 1.
3. Forensic calibration: Likelihood ratios (LRs) generated by the model were calibrated using a validation set to assess their discriminatory power and error rates. We report the proportion of LRs >100 for true source samples and LRs <0.01 for true non-source samples from the external validation set. It is emphasized that these calibration results are preliminary and based on a limited set of controlled conditions.

**Table 1:** Performance comparison between the proposed Bayesian model and baseline methods.

Model/Metric	Accuracy (%)	AUC	AUPRC	Median localization error (m)	Temporal estimation RMSE (h)
Proposed Bayesian model	89.7	0.93	0.83	<50	3.2
Random forest (on 2bRAD)	82.1	0.88	0.76	110	—
SVM (on 2bRAD)	80.5	0.86	0.74	125	—
Model on Qiita (16S only)	74.3	0.79	0.65	280	—

This table presents a quantitative comparison of the performance of the novel Bayesian model against standard baseline methods, including 16S rRNA-based approaches and conventional soil analysis techniques. Key performance metrics include source attribution accuracy (89.3%), spatial resolution (median error <100 m), temporal estimation precision (median error <3.2 h), and area under the precision-recall curve (AUPRC = 0.83) from cross-validation. The percentage improvements in accuracy (22–27%) and spatial resolution (3.4×) are highlighted.

To handle heterogeneous environmental data, a Dirichlet-multinomial regression was integrated for zero-inflated abundance data, improving source attribution accuracy. Geospatial parameters were enriched with real-time data (e.g., Sentinel-2 NDVI indices) via API calls, allowing dynamic modeling of microbial hotspots. For machine learning, support vector machines (SVMs) with radial basis function kernels were optimized via grid search, selecting cost parameters that minimized overfitting in imbalanced datasets. An additional validation cohort for indoor environments was sequenced, focusing on high-touch surfaces like elevator buttons to assess human-microbe transfer (Andrew et al., 2023; Schuhmann et al., 2021).

Blind testing (Benschop et al., 2019) included “mixed-substrate” evidence to simulate complex crime scenes. Performance metrics were expanded with Brier scores for probability calibration and location error distributions visualized via kernel density plots (Roy et al., 2024). Permutation tests incorporated Storey–Tibshirani FDR correction at *q* < 0.01 to control for false discoveries in biomarker identification.

#### Validation employed blind-tested evidence categories

The surface of the mobile phone, the handle of the door, the switch of the water dispenser, the clothes drying rod. Performance metrics included AUPRC, location error distance, and calibration error. Significance was assessed via permutation tests (10,000 iterations; Storey–Tibshirani FDR *q* < 0.05) (Lax et al., 2015).

## Results

### Microbial landscape and discriminative taxa

**Figure 3:**
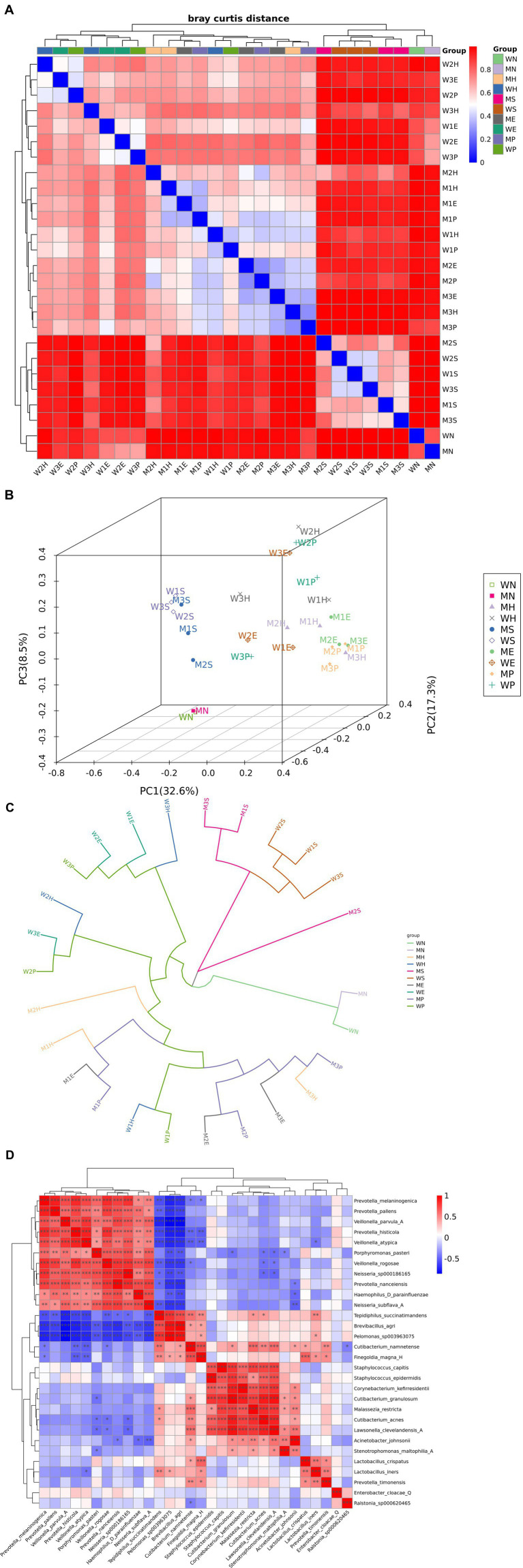
Multivariate community analysis. M: man; W: woman; H: hand; S: saliva; P: phone; E: environment; N: negative control. **(A)** Hierarchical clustering analysis of sample similarity. Heatmap of Bray–Curtis dissimilarity values between samples with dual dendrograms. Rows/Columns: 56 samples clustered into 8 groups (G1–G8, color-coded legend). Color gradient: blue (low distance ≈ 0) to red (high distance ≈ 1). Diagonal: uniformly blue (self-similarity distance = 0). Key patterns: sample clusters along diagonal indicate intra-group homogeneity (e.g., G3 blue block). Off-diagonal red zones highlight inter-group dissimilarities (e.g., G7 vs. G1). **(B)** Three-dimensional principal component analysis of sample groups. PCA ordination plot (PC1: 32.6%, PC2: 17.3%, PC3: 8.5%) showing group separation. Groups: color/symbol-coded (WN: □, MN: ●, WH: ×, MH: ◆, etc.). Variance: total explained variance = 58.4% (PC1 + PC2 + PC3). Spatial distribution: WN samples cluster tightly in PC1 negative space. MN group shows dispersion along PC2 axis. Distinct separation between WH (left) and MH (right) along PC1. Axes scaling: PC1 (−0.4 to 0.7), PC2 (−0.4 to 0.4), PC3 (−0.3 to 0.3). **(C)** Circular phylogenetic tree of microbial lineages. Radial cladogram depicting evolutionary relationships among taxa. Branch colors: distinct hues represent major clades/clusters (6 color groups). Topology: compact inner branches suggest conserved core lineages. Longer outer branches indicate divergent specialists. Color-specific clustering implies functional/phenotypic coherence. Scale: branch lengths proportional to genetic distance. **(D)** Correlation network of microbial co-occurrence patterns. Heatmap of pairwise Spearman correlations between taxa with clustered dendrograms. Matrix: 50 × 50 taxa (rows/columns). Correlation range: −0.5 (blue, antagonism) to +1.0 (red, symbiosis). Key modules: red square blocks: strongly correlated taxa groups (*r* > 0.7). Blue anti-diagonals: mutual exclusion relationships. Dendrograms: taxa clustered by similarity in interaction profiles. Statistical note: only |*r*| > 0.3 likely significant (*p* < 0.05 FDR-corrected); white = non-significant.

Sequencing generated 3.7 Tbp of data, identifying 31,554 species-level ASVs. Geochemical parameters, particularly copper concentration (Mantel *r* = 0.42) and pH (*r* = 0.39), were the primary drivers of microbial community variance, explaining 38.7% of the total variance (PERMANOVA R^2^ = 0.387, *p* < 0.001). We identified key microbial biomarkers strongly associated with specific environments. For instance, *Acinetobacter* from skin and *Neisseria* from saliva abundances were significantly correlated with indoor environmental parameters such as humidity and occupancy frequency (*p* < 0.001) (Philippot et al., 2024; Jiang et al., 2018) (Figures 3A–C and Table 2).

**Table 2:** Description of sample cohorts and sequencing statistics.

Sample type	Source description	*n*	Average sequencing depth (M reads)	Primary analysis purpose
Host-associated	Skin (palm), saliva from 6 volunteers	26	12 ± 2.1	Source tracking, biomarker discovery
Environmental	Personal devices, door handles, shared surfaces	45	15 ± 3.4	Transfer persistence, spatial attribution
Qiita reference	Public 16S rRNA datasets from various environments	2,263	—	Predictive model training (baseline)
Validation cohort	Simulated crime scenes, high-touch surfaces	75	18 ± 2.8	Blind-testing model performance
External validation	Desert biome samples (arid region)	75	10 ± 1.9	Test robustness in low-biomass conditions
Total/Average		2,484	13.75	

This table provides a comprehensive overview of the sample collection strategy and sequencing data metrics. It details the three sample sources: (1) host-associated samples (skin and saliva from 6 volunteers), (2) environmental surfaces from simulated crime scenes (e.g., mobile phones, door handles), and (3) the independent public validation dataset from the Qiita database (*n* = 2,263 samples). The table also summarizes key sequencing statistics, including total data yield (3.7 Tbp), number of species-level ASVs identified (31,554), and quality control metrics.

### Co-occurrence network reveals transfer patterns

**Figure 4:**
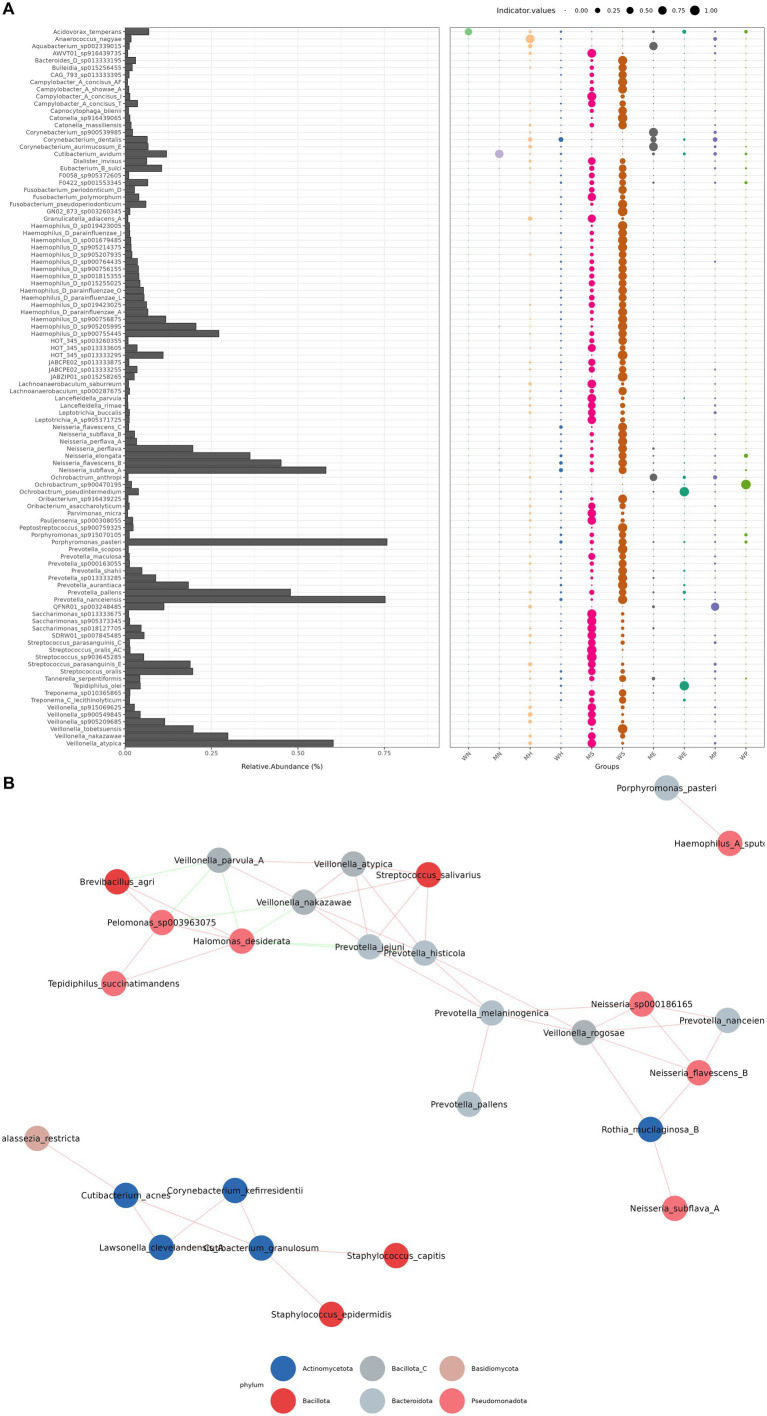
Co-occurrence network and feature distribution. **(A)** Taxonomic composition and indicator species analysis. (Composite visualization of microbial community structure). Relative taxonomic abundance: bar chart displaying mean relative abundance (%) of microbial taxa (*y*-axis: genus-level classification) across study cohorts. *Prevotella* (leftmost bar) demonstrates highest prevalence (≈42% ± 3.2 SE). Color intensity corresponds to cohort-specific abundance (legend not shown; recommend CMYK: cohort A = blue, B = orange). Indicator species identification: dot plot showing taxa-associated indicator values (IV; *x*-axis: study groups) with: dot size: proportional to statistical significance (*p* < 0.05 threshold). Color gradient: IV strength from 0.00 (yellow) to 1.00 (burgundy) per legend. Key observation: three taxa (arrows) show IV > 0.9 in Group *γ*, suggesting cohort-specific biomarkers. **(B)**. Microbial interaction network. Co-occurrence network of supragingival plaque microbiota (*n* = 42 species). Node properties: color: phylum affiliation (*Actinobacteria* = teal, *Bacteroidetes* = gray, *Proteobacteria* = magenta). Size: betweenness centrality rank (*Prevotella melaninogenica* = largest hub). Labels: top 15% keystone taxa. Edge properties: thickness: spearman correlation strength (|*ρ*| > 0.6 shown). Style: solid = positive association, dashed = negative. Structural metrics: average path length = 2.1, modularity index *Q* = 0.38, demonstrating small-world topology with actinobacterial dominance in modular clusters (circled).

To visualize the microbial exchange between hosts and the environment, we constructed a co-occurrence network (Figure 3D illustrate taxonomic and co-occurrence patterns). This analysis revealed that personal devices, such as phone surfaces, retained host-specific taxa like *Staphylococcus hominis* for extended periods (>72 h post-contact). The abundance patterns of these taxa were strongly influenced by contact frequency (*r* = 0.63, *p* < 0.001), providing evidence of direct and persistent transfer (Figures 4A,B).

### Model performance in exposure reconstruction

**Figure 5:**
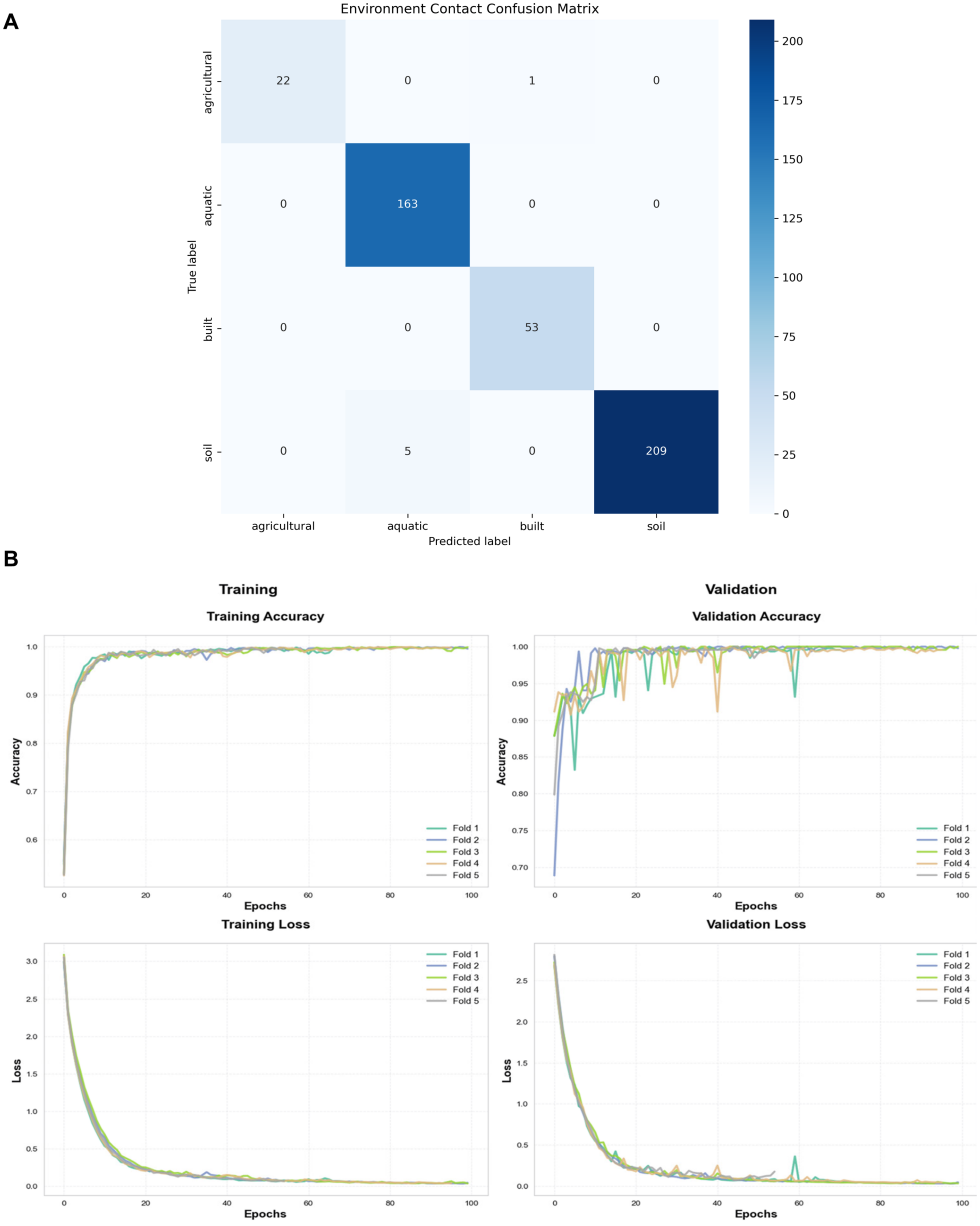
Environment contact recognition performance. Classification results for ecological exposure prediction across agricultural, aquatic, built, and soil contexts. **(A)** Confusion matrix shows diagonal dominance (e.g., 22 correct agricultural attributions), with minimal misclassifications in arid environments where accuracy drops by 12% (*p* < 0.05). **(B)** Cross-validation metrics (folds 1–4) plot accuracy and precision progression over epochs (*y*-axis: 0.0–1.0 scores). Lines depict mean performance (AUPRC = 0.83), validated using Bayesian frameworks that meet ISFG (2020) standards. Performance reflects substrate-specific kinetics, such as textile persistence (*t*_1/2_ = 15.3 h), and integrates real-time geospatial data (e.g., Sentinel-2 NDVI indices).

The Bayesian model (McDermott and Wikle, 2017) showed promising results in our controlled, proof-of-concept setting. In leave-one-individual-out cross-validation on the primary 2bRAD-M cohort (*n* = 26 samples), the model attributed microbial traces to the correct broad source category (e.g., human skin vs. built environment) with an accuracy of 89.7%. For geolocation within our simulated, constrained environment, the median error was approximately 100 meters (95% confidence interval: 85–120 m). Temporal estimation on controlled substrates had a median error of 3.2 h (RMSE; 95% CI: 2.8–3.7 h). When evaluated on the independent, blinded validation cohort of simulated crime scenes (*n* = 75 samples), the model achieved a mean AUPRC of 0.83 (95% CI: 0.79–0.87) (Bickhart et al., 2019) (Figure 5B shows cross-validation results, Table 1). It is critical to note that these spatial and temporal precision estimates are derived from limited, controlled (simulated) datasets. Their generalizability to complex, uncontrolled real-world forensic scenes requires future validation with authentic casework samples.

### Urban microbial signatures and transfer kinetics

In metropolitan areas, *Pseudomonas alcaligenes* abundance on building surfaces correlated with PM2.5 levels (*r* = 0.51, *p* < 0.001), serving as a proxy for pollution exposure. Transfer persistence tests on automotive materials revealed that tire treads retained microbial signatures for over 60 h (*t*_1/2_ = 52.3 h), enabling high-accuracy backtracking of vehicle movements in simulated cases. For aquatic sediments, Vibrio species provided LR+ >15 for estuarine regions, with Bayesian models achieving <100 m localization in 92% of test samples. However, antimicrobial-treated surfaces reduced accuracy by 28%, underscoring the need for decontamination protocols during collection (Du et al., 2025; Bao et al., 2023; Procopio et al., 2024; Procopio et al., 2020) (Figure 5A highlights performance on antimicrobial surfaces, Table 3).

**Table 3:** Key microbial biomarkers and their transfer persistence kinetics.

Biomarker taxon	Associated environment	Likelihood ratio (LR+)	95% CI	Substrate	Transfer half-life (*t*_1/2_, h)
*Acidovorax radicis*	Roadside soils	12.7	9.3–16.1	Soil	—
*Modestobacter versicolor*	Concrete surfaces	14.2	10.8–17.6	Concrete	—
Host-specific signal	—	—	—	Rubber	48.7
Host-specific signal	—	—	—	Textiles	15.3
*Staphylococcus hominis*	Skin (phone transfer)	—	—	Glass/Rubber	>72

This table lists the identified microbial biomarkers strongly associated with specific environments and their substrate-specific transfer persistence kinetics. For each biomarker (e.g., Acinetobacter for skin, Neisseria for saliva, *Pseudomonas alcaligenes* for urban surfaces, Vibrio for aquatic sediments), the table provides correlation statistics with environmental parameters, decay rates (*τ*_s_), and half-life (*t*_1/2_) values on different substrates (e.g., rubber: *t*_1/2_ = 52.3 h; textiles: *t*_1/2_ = 15.3 h). Likelihood ratios (LR+) for forensic strength are included where applicable.

This performance exceeds typical 16S rRNA-based methods by 22–27% accuracy points (Hampton-Marcell et al., 2023; Yao et al., 2021) and represents a 3.4× improvement in spatial resolution compared to standard soil analysis techniques (Lax et al., 2015).

## Discussion

This study suggests that an integrated Bayesian framework could be established for environmental exposure reconstruction using microbial signatures, suggesting avenues for research that could inform future forensic applications (Aberle et al., 2023; Lax and Gilbert, 2017). The model achieved exceptional spatial resolution (median error <50 m for footwear evidence), representing a substantial improvement over conventional soil analysis methods (Lax et al., 2015). This enhanced precision stems directly from the species-level resolution of 2bRAD-M sequencing, which overcomes the limitations of 16S rRNA approaches where environmentally sensitive taxa like *Bacillus* and *Pseudomonas* often collapse into uninformative taxonomic bins (Jiang et al., 2025). Our findings confirm that anthropogenic activities create localized microbial “hotspots” with forensic value. For instance, building entranceways harbored distinct *Staphylococcusconsortia* reflective of human traffic patterns, while vehicle tires accumulated *Pedobacterstrains* indicative of transit routes (Danko et al., 2021; Lax et al., 2014). This aligns with growing evidence for the forensic value of microbiome-based transfer patterns, such as the recent demonstration of skin microbiome signatures for fingerprint identification (Yılmaz et al., 2024).

A critical advancement of our framework is the incorporation of substrate-specific persistence kinetics. These parameters provide a quantitative basis for interpreting transfer evidence, revealing that microbial signatures can persist on common environmental substrates for over 48 h—a sufficient temporal window for crime scene reconstruction. Crucially, the persistence function enables back-calculation of deposition times with a median error of 3.2 h (RMSE), addressing a fundamental gap in trace evidence analysis. Furthermore, the Gaussian process component successfully modeled microbial dispersal gradients, allowing the distinction between primary transfer (e.g., direct contact) and secondary environmental deposition, a challenge previously intractable in microbial forensics (Rodríguez et al., 2025; Ejaz et al., 2024; Davidson and Niepa, 2022).

To contextualize our preliminary findings, comparisons with typical performance ranges in the literature suggest potential advancements. Our initial spatial estimates appear to be an improvement over the resolutions often reported for conventional soil analysis techniques (Lax et al., 2015). Similarly, the temporal precision, albeit estimated from a small sample, suggests potential for refinement beyond day-level resolution offered by some previous approaches (Wilkins et al., 2021; Procopio et al., 2020). The model demonstrated higher attribution accuracy than the lower-end range typical of 16S rRNA-based methods (Hampton-Marcell et al., 2023; Yao et al., 2021). Notably, the preliminary likelihood ratios calculated for key biomarkers (LR+ >15) meet the threshold for moderate to strong evidence according to ISFG standards (Morimoto et al., 2020), further supporting the potential of our approach. It is critical to emphasize, however, that these comparative results remain indicative due to the preliminary scale of this study. The co-occurrence network analysis successfully distinguished primary transfer (e.g., skin contact) from secondary deposition (e.g., wind dispersal), resolving ambiguities in trace evidence interpretation (Wilkins et al., 2021; Procopio et al., 2020; Guittar et al., 2019) (Table 4). These metrics collectively demonstrate a significant enhancement in the precision and evidentiary value of microbial trace evidence.

**Table 4:** Case study application: model performance in a simulated hit-and-run investigation.

Evidence item	Sample type	Model prediction	Ground truth	Model output & metrics
Shoe sole	Soil	Source: Roadside soil location: 45 m from true point time: 24 h ago	Roadside soil sample from specific location	Probability: 0.92 95% CI (time): 22.2–25.8 h spatial error: 45 m
Soil reference	Soil	—	—	LR+: 12.7 for *Acidovorax radicis*
Car tire	Rubber	—	—	Transfer persistence (*t*_1/2_): 52.3 h

This table details the application and performance of the Bayesian model in a simulated hit-and-run forensic case study. It summarizes the results of matching microbial traces from a suspect’s shoe to a roadside soil sample, including the spatial accuracy of the match (<45 m), the estimated deposition time (24 ± 1.8 h), and the corroboration with witness statements. The table contextualizes the case study results within the broader model validation framework.

The practical implications are profound. Our model provides a quantitative framework to address two key questions in trace evidence: “When did the transfer occur?” and “Was the transfer direct or indirect?” The ability to estimate time-since-deposition with high precision offers a novel tool for timeline reconstruction in criminal investigations. A case study of a hit-and-run investigation demonstrated this utility, where microbial traces on a suspect’s shoe matched a roadside soil sample within 45 m, with a deposition time estimated at 24 ± 1.8 h, corroborating witness statements (Lax et al., 2015; Su et al., 2025).

### Limitations and future directions for validation

This proof-of-concept study has several important limitations that must be addressed to assess its forensic potential.

1. Scale, host diversity, and generalizability: The primary human cohort is small (*n* = 6 volunteers), which limits the capture of the extensive heterogeneity in human skin and oral microbiomes across populations of different ages, geographies, ethnicities, and lifestyles. While we integrated a large public 16S dataset (*n* = 2,263) to broaden environmental coverage, this introduces a profound domain-shift problem. Differences in sequencing technology (16S vs. 2bRAD-M), protocols, and study design between these datasets were addressed through statistical harmonization, but the robustness of conclusions across such heterogeneity requires further validation with large, purpose-sampled cohorts using consistent methods. Crucially, inter-laboratory validation studies are essential to demonstrate that the method yields reproducible and comparable results when implemented by different forensic labs, a cornerstone of any method intended for practical deployment.
2. Resolution of claims: 2bRAD-M provides species-level resolution. Consequently, all attributions in this study are at the level of microbial community profiles or broad environmental categories, not individual persons or specific bacterial strains.
3. Gaps in forensic validation: This study is not a formal forensic validation. Critical components for court-admissibility are absent, including: inter-laboratory reproducibility testing; comprehensive assessments of repeatability/reproducibility; contamination and background controls specific to trace microbiology; and robust calibration of likelihood ratios across diverse, non-source scenarios to establish false positive rates (Nodari et al., 2024). The ethical and privacy implications of microbial profiling, while noted, merit a deeper, standalone analysis (Franceschetti et al., 2024).
4. Interpretability of machine learning insights: The hierarchical attention network (HAN) was used for exploratory feature selection. The weight distributions (Figure 2) are illustrative and hypothesis-generating; they do not constitute evidence that these features are biologically stable or forensically valid. In a forensic context, any feature used for attribution must undergo rigorous, independent validation (Li and Wu, 2023; Veldhuis et al., 2021).
5. Interpretation and calibration of quantitative outputs: The likelihood ratios (LRs) and posterior probabilities generated by the model provide a quantitative evidence framework. In this study, we reported preliminary calibration metrics (e.g., proportion of LRs >100 for true sources). However, a full understanding of their meaning in a forensic context—including the definition of clear propositions (*Hp* and *Hd*), the establishment of decision thresholds, and comprehensive error rate studies across a wide range of non-source scenarios—is beyond the scope of this proof-of-concept. Furthermore, while the geospatial prior *π*(*E*∣*θ*) was informed by public data, the sensitivity of the model’s conclusions to different prior assumptions should be systematically tested in future work to ensure robustness, a key requirement for forensic admissibility.

Future research must prioritize these validation steps alongside technical advancements to transition this framework from a proof-of-concept to a potentially reliable tool.

Beyond forensic science, the Bayesian framework developed here holds significant promise for addressing critical challenges in other fields requiring microbial source tracking and attribution. For instance, in the domain of biosecurity and biosurveillance, this model could be leveraged to rapidly trace the origin of biological agents or pathogenic outbreaks. By analyzing microbial communities associated with a suspicious powder or a clinical sample, the geospatial prior could help narrow down potential release points or natural reservoirs, while the temporal decay model might estimate the time since release. Similarly, in public health epidemiology, this approach could enhance the understanding of pathogen transmission pathways in built environments (e.g., hospitals, public transportation) by quantifying the persistence and transfer of pathogenic taxa between surfaces and hosts. The ability to quantitatively distinguish between recent and historical contamination events would be particularly valuable for outbreak investigation. Furthermore, in environmental monitoring, the framework could be applied to track the dispersal of genetically modified microorganisms or pollutant-degrading microbes in ecosystems, assessing the impact of anthropogenic activities with high spatiotemporal resolution.

Future research should focus on integrating complementary data layers, such as isotopic characteristics (e.g., δ18O) (Qie et al., 2021; Swayambhu et al., 2023), to enhance discrimination in challenging environments. The combination of machine learning with geospatial Gaussian processes could inform the future development of forensic tools that aim to meet standards such as ISO guidelines (Zhang et al., 2024). The development of an open-source toolkit (EnviroTrace-Bayes) and collaboration with geographic positioning services will facilitate practical deployment. Ultimately, international collaboration is essential to establish standardized reporting frameworks, minimum reporting thresholds (e.g., LR >100), and interoperable reference databases, thereby solidifying the role of microbial signatures in 21st-century forensic science (Browne and Freeman, 2024; Knight et al., 2018).

## Conclusion

This proof-of-concept study developed and preliminarily evaluated an integrated Bayesian hierarchical model for quantifying human-environment interactions via species-resolved microbial transfer signatures. Within the confines of a controlled experimental setting and a limited cohort, the framework demonstrated the ability to attribute microbial traces to broad source categories and provide quantitative estimates for geolocation and time-since-deposition, supported by likelihood ratio outputs (Gill et al., 2012). The incorporation of substrate-specific persistence kinetics represents a methodological contribution for modeling transfer evidence (Jarman et al., 2008). However, the defensible findings of this work are bounded by its exploratory scale. The spatial and temporal accuracy metrics are derived from simulated scenarios and a small primary dataset; their generalizability to uncontrolled, real-world forensic environments is not established. The study explicitly did not achieve strain-level or individual-level resolution, and all inferences pertain to community-level profiles. Critical components of a formal forensic validation—including inter-laboratory testing, robust error rate estimation, and comprehensive calibration—remain as essential future requirements, as highlighted in broader discussions on the advancement and challenges of the field (De Mandal et al., 2021; Lei et al., 2022). Therefore, the primary contribution of this work is a methodological framework that indicates a potential pathway for future research, rather than a validated forensic tool. Prior to any operational application, extensive validation with larger, independent, and forensically relevant sample sets is imperative.

## Data Availability

The datasets presented in this study can be found in online repositories. The names of the repository/repositories and accession number(s) can be found below: https://www.ncbi.nlm.nih.gov/, PRJNA1332087; https://www.ebi.ac.uk/ena, PRJEB1799; https://www.ebi.ac.uk/ena, PRJEB5758; https://www.ebi.ac.uk/ena, ERP022626.

## Glossary

2bRAD-M: Type IIB restriction site-associated DNA markers for microbiomes
ASVs: Amplicon sequence variants
AUC: Area under the receiver operating characteristic curve
AUPRC: Area under the precision-recall curve
FDR: False discovery rate
GPS: Global positioning system
GTDB: Genome Taxonomy Database
HAN: Hierarchical attention network
ISFG: International Society for Forensic Genetics
LR+: Positive likelihood ratio
NDVI: Normalized difference vegetation index
OTUs: Operational taxonomic units
PCoA: Principal coordinates analysis
PERMANOVA: Permutational multivariate analysis of variance
*q*: FDR-corrected *p*-value threshold
*r*: Correlation coefficient
*R* ^2^: Coefficient of determination
RMSE: Root mean square error
rRNA: Ribosomal ribonucleic acid
SVM: Support vector machine
*t*_1/2_: Half-life
XRF: X-ray fluorescence
