# Supplementary figures and images for: Quantifying human-environment interactions through Bayesian modeling of species-resolved microbial transfer signatures: an exploratory proof-of-concept study
Source: Front Microbiol. 2026 Mar 24;17:1781392. doi: 10.3389/fmicb.2026.1781392 (PMC13053512; doi:10.3389/fmicb.2026.1781392)

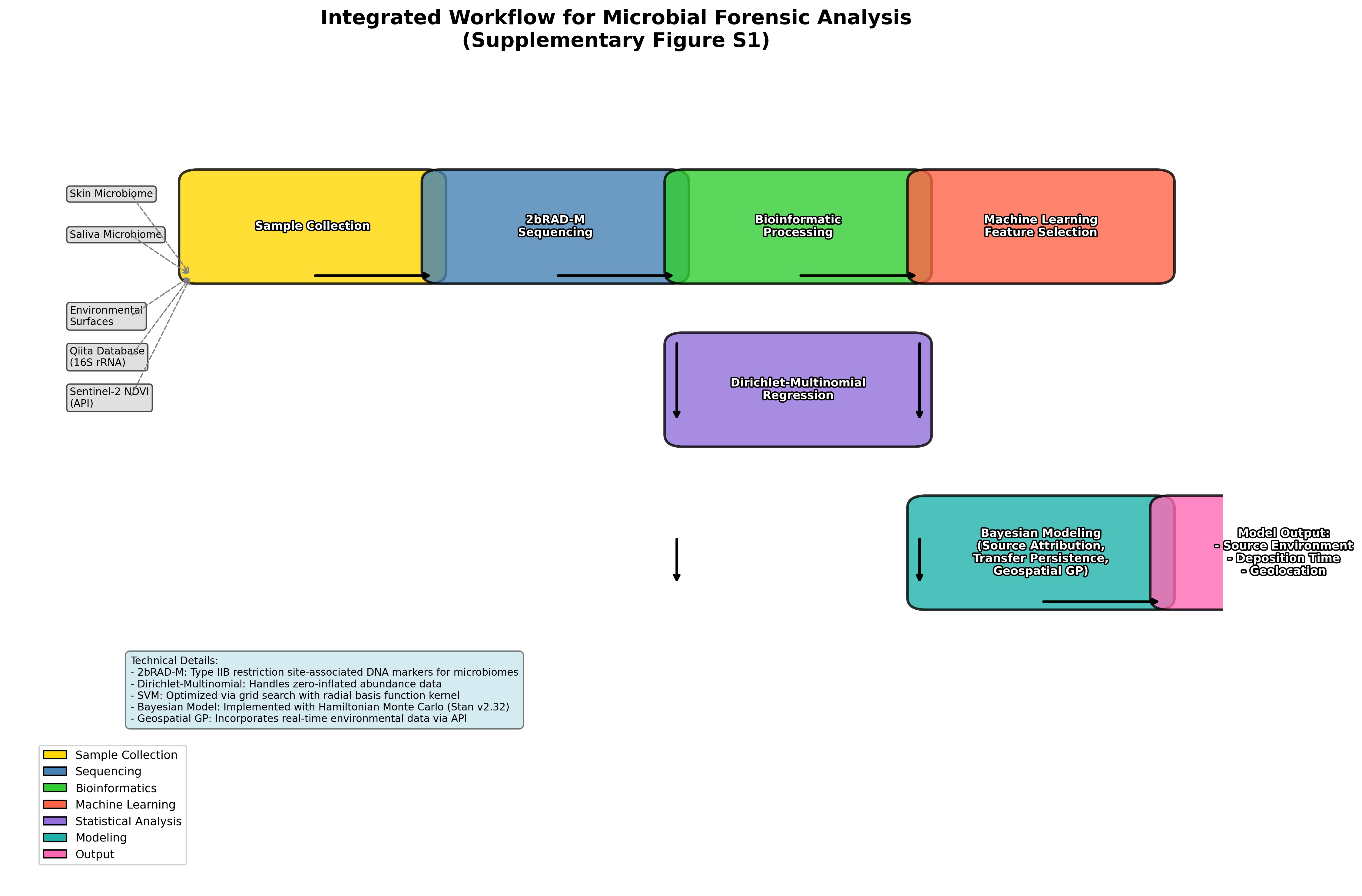

Supplement: Supplementary file 1 [file Image_1.JPEG]
